# Cu-Doped ZnO Nanoparticles for Non-Enzymatic Glucose Sensing

**DOI:** 10.3390/molecules26040929

**Published:** 2021-02-10

**Authors:** Amira Mahmoud, Mosaab Echabaane, Karim Omri, Julien Boudon, Lucien Saviot, Nadine Millot, Rafik Ben Chaabane

**Affiliations:** 1 Laboratory Interfaces and Advanced Materials (LIMA), Faculty of Science of Monastir, University of Monastir, 5019 Monastir, Tunisia; Amira_Mahmoud@etu.u-bourgogne.fr (A.M.); Rafik.benchaabane@fsm.rnu.tn (R.B.C.); 2 Laboratoire Interdisciplinaire Carnot de Bourgogne (ICB), UMR 6303 CNRS, Université Bourgogne Franche-Comté, 9 av. A. Savary, BP 47870, 21078 Dijon, France; Julien.Boudon@u-bourgogne.fr (J.B.); Lucien.Saviot@u-bourgogne.fr (L.S.); 3 NANOMISENE Lab., LR16CRMN01, Centre for Research on Microelectronics and Nanotechnology CRMN of Technopark of Sousse, B.P. 334, Sahloul, 4034 Sousse, Tunisia; mosaab.echabaane@gmail.com; 4 Laboratory of Physics of Materials and Nanomaterials Applied at Environment (LaPhyMNE), Faculty of Sciences of Gabes, University of Gabes, 6029 Gabes, Tunisia; omrikarim16@gmail.com

**Keywords:** ZnO, Cu doping, nanoparticles, electrochemistry, glucose, non-enzymatic sensor

## Abstract

Copper-doped zinc oxide nanoparticles (NPs) Cu*_x_*Zn_1−*x*_O (*x* = 0, 0.01, 0.02, 0.03, and 0.04) were synthesized via a sol-gel process and used as an active electrode material to fabricate a non-enzymatic electrochemical sensor for the detection of glucose. Their structure, composition, and chemical properties were characterized using X-ray diffraction (XRD), transmission electron microscopy (TEM), Fourier-transform infrared (FTIR) and Raman spectroscopies, and zeta potential measurements. The electrochemical characterization of the sensors was studied using cyclic voltammetry (CV), electrochemical impedance spectroscopy (EIS), and differential pulse voltammetry (DPV). Cu doping was shown to improve the electrocatalytic activity for the oxidation of glucose, which resulted from the accelerated electron transfer and greatly improved electrochemical conductivity. The experimental conditions for the detection of glucose were optimized: a linear dependence between the glucose concentration and current intensity was established in the range from 1 nM to 100 μM with a limit of detection of 0.7 nM. The proposed sensor exhibited high selectivity for glucose in the presence of various interfering species. The developed sensor was also successfully tested for the detection of glucose in human serum samples.

## 1. Introduction

Nanoscale semiconducting metal oxides have been triggering extensive research activities owing to their unique electronic, mechanical, and optical properties [1]. Their good crystallinity and large surface-to-volume ratio make them good candidates for producing highly sensitive chemical sensors [2]. In particular, nanoparticles (NPs) made of ZnO, a wide band-gap semiconductor, are attracting a lot of attention due to their low-temperature synthesis, various morphologies, high crystallinity, good optical properties, and excellent electrical characteristics. ZnO has a broad range of applications in photocatalysis [3], photovoltaics [4], gas sensing [5], electronics (diodes), and water-splitting [6]. It has also received growing interest in the field of biochemical sensors [7] thanks to its outstanding sensitivity to chemical species. NPs have a large surface-to-volume ratio. This improves their sensing properties compared to flat ZnO surfaces by multiplying the interactions at the molecular level with surrounding chemical species [8]. Furthermore, the high isoelectric point (IEP) of ZnO [9] allows its surface to adsorb biocatalysts with a low IEP via electrostatic interactions. In addition, its unique physico-chemical behavior, high electron transfer capability, and high reactivity with oxygen make ZnO one of the most promising semiconductor metal oxides for many applications and have led to rapid progress in the development of (bio)chemical sensors [10].

Despite the outstanding properties of ZnO nanostructures, the sensing capability of pure ZnO was found to be inadequate [11]. Recent studies have shown that the combination of ZnO nanostructures with catalyst species (noble metals, transition metals, carbon nanotubes, graphene, etc.) can overcome this issue. These various catalyst species allow for the creation of ZnO-based nanostructures with a large surface area for catalysis, short diffusion pathways for charge carriers, and efficient electron transport. Doping ZnO NPs with transition metals, such as Al [12], Mn [13], Co [14], Ni [15], and Fe [16], has revealed a significant impact on the electrical, optical, and magnetic properties that might enhance the sensing response [17]. Cu is frequently used as a doping transition metal to develop ZnO NPs with improved properties for advanced applications. Indeed, Cu atoms can substitute Zn in the ZnO lattice structure thanks to their identical valence state and similar ionic radii [18]. In addition, Cu has a large ionization energy and low formation energy, which speeds up the integration of Cu into the ZnO lattice [19]. The incorporation of Cu into ZnO provides a high electronic conductivity and good donor defects. This facilitates the charge separation and improves the transport properties in ZnO nanostructures [20].

Despite quite similar radii for Cu^2+^ and Zn^2+^ cations (Cu^2+^: 0.73 Å, Zn^2+^: 0.74 Å) [18], relatively low values of Cu content can be incorporated in the ZnO wurtzite structure, without the phases demixing. In the case of Cu-doped ZnO nanorods synthesized using an ice-bath-assisted sonochemical technique, Othman et al. [21] reported a single phase (wurtzite) up to a doping content of 4% Cu. For higher Cu content, a CuO secondary phase appeared, which generated distortions in the ZnO host lattice. Similar behavior was reported by Lee et al. [22], who synthesized Cu-doped ZnO using a sol-gel method, and by Chandekar et al. [23], who prepared Cu-doped ZnO NPs using the auto combustion method. Therefore, mixed phases (metallic copper, copper oxides, or any binary zinc copper phase) mostly appear for 5% Cu doping.

Sensing with Cu-based nanomaterial is extremely sensitive thanks to its good electrochemical activity and proper redox potentials. Cu is an efficient electrical conductor at a low overpotential. The large surface area and chemical properties of Cu NPs provide high adsorption rates and strong interactions with analytes [24]. Cu NPs also possess catalytically active sites with enhanced electron transfer rates [25].

Recently, glucose biochemical sensors based on ZnO nanostructures have attracted considerable interest. Glucose is a critical component in the blood that provides energy for living organisms. Abnormal levels of glucose are the source of multiple medical disorders. They play a major role in the initiation and development of diabetes mellitus, one of the major causes of death in the world [26]. Regulating and controlling glucose is therefore a major health issue. Improving glucose-sensing devices with fast, reliable, and sensitive recognition of the glucose levels has become an absolute requirement in order to meet the needs of diabetes patients and also for other non-medical areas of interest, including biotechnology and food-processing industries [27].

The electrochemical approach stands out among the various possible techniques that are suitable for glucose determination [28]. Two main kinds of glucose sensors are used in electrochemical technology: enzymatic-based sensing and non-enzymatic glucose sensing [29,30]. Most of the previous glucose sensors relied on enzymes to electrocatalyze the oxidation of glucose [31]. The advantages of enzymatic glucose biosensors are their excellent selectivity and high sensitivity. However, they present drawbacks, such as poor vitality and reduced chemical and thermodynamic stability, which originate from the intrinsic nature of enzymes. In addition, their high cost, complicated immobilization procedures, rigorous operating conditions (appropriate temperature, pH, humidity, and toxic chemicals), and poor reproducibility further limit their development [32,33]. To overcome these shortcomings, non-enzymatic glucose sensors are being considered due to their potential advantages, including a simple fabrication process, improved stability under different conditions, reproducibility, and low cost [34].

In a previous study [35], we reported the development of pure and copper-doped ZnO NPs (one composition) as a form of non-enzymatic glucose detection. Herein, we study the impacts of varying Cu doping concentration on the physicochemical properties of ZnO NPs for improved non-enzymatic glucose sensing. The influence of Cu doping on the structural, morphological, and chemical properties of ZnO NPs was studied in detail using X-ray diffraction (XRD), transmission electron microscopy (TEM), zeta potential measurements, and Fourier-transform infrared (FTIR) and Raman spectroscopies. We developed a procedure to coat the NPs on indium-doped tin oxide (ITO) to fabricate sensing electrodes. The electrochemical properties of the Cu*_x_*Zn_1−*x*_O NP-based electrodes were studied using cyclic voltammetry, electrochemical impedance spectroscopy, and differential pulse voltammetry. We were thus able to identify the optimal Cu content that presented outstanding features, such as high sensitivity, selectivity, a wide linear range, and a low limit of detection. These excellent performances, together with the easy fabrication process, make this approach promising for developing low-cost, non-enzymatic electrochemical sensors for the determination of glucose contents.

## 2. Results and Discussion

### 2.1. Structural and Morphological Characterization and Chemical Studies of Cu_x_Zn_1−x_O NPs

**Figure 1:**
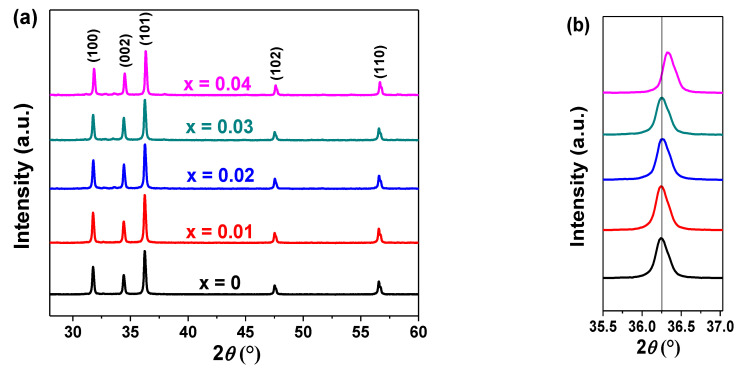
(**a**) X-ray diffraction (XRD) patterns of Cu*_x_*Zn_1−*x*_O nanoparticles (NPs) (*x* = 0, 0.01, 0.02, 0.03, and 0.04) and (**b**) the enlarged view of the (101) peak.

**Figure 2:**
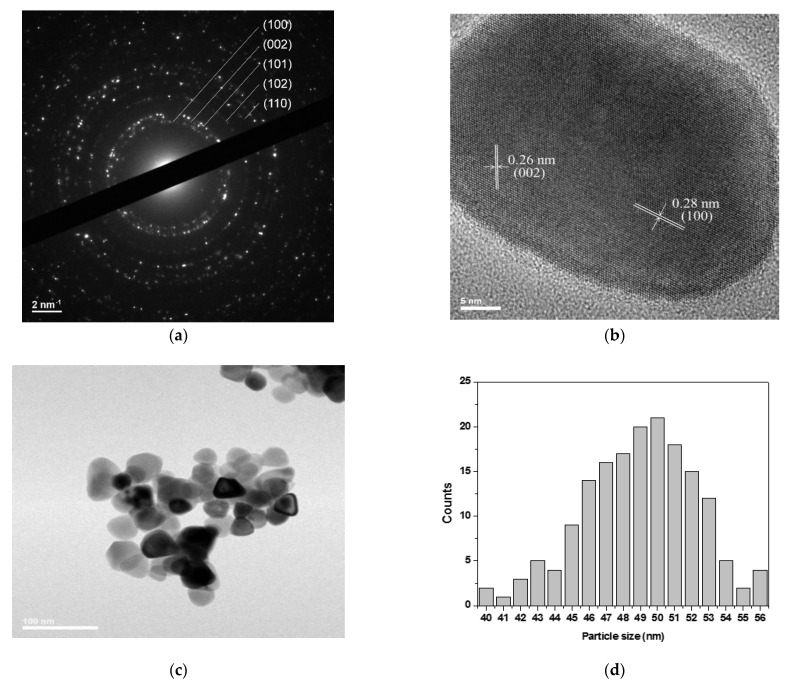
(**a**) Selected area electron diffraction (SAED) pattern (40 cm camera length), (**b**) high-resolution transmission electron microscopy (HRTEM) image, (**c**) TEM image, and (**d**) size distribution histogram of Cu_0.04_Zn_0.96_O NPs.

The X-ray diffraction patterns of Cu*_x_*Zn_1−*x*_O NPs (*x* = 0, 0.01, 0.02, 0.03, and 0.04) are shown in Figure 1. The (100), (002), (101), (102), and (110) Bragg peaks matched with the hexagonal phase of ZnO (International Centre for Diffraction Data (ICDD) 36-1451). These peaks were sharp and intense, which proved the high crystallinity of the NPs as also highlighted by the SAED (selected area electron diffraction) pattern and high-resolution transmission electron microscopy (HR-TEM) analysis (Figure 2a,b). In Figure 2a, the diffraction rings were relatively continuous, in agreement with the nanosize of the crystallites. The rings of the Cu_0.04_Zn_0.96_O sample could be indexed to the (100), (002), (101), (102), and (110) planes of hexagonal wurtzite ZnO. The HR-TEM image (Figure 2b) clearly depicts the presence of aligned lattice fringes with interplane spacings of 0.28 nm and 0.26 nm, corresponding to the (100) and (002) lattice planes of wurtzite ZnO, respectively. Hence, the SAED pattern and HR-TEM analysis were in good accordance with the XRD. For the doped samples, no new diffraction peaks related to possible Cu-rich phases were observed in the XRD patterns. This proves that the Cu ions were completely incorporated into the ZnO lattice and did not cause a change in the structure since the doping concentration was below the solubility limit [36]. It is noteworthy to mention that when the Cu doping was increased to *x* = 0.05, the XRD patterns of the NPs presented extra peaks related to the Cu segregated phases (see Supplementary Information, Figure S1).

Furthermore, a shift in the peak position was detected as the copper concentration increased (Figure 1b). This shift toward a higher 2*θ* was easily distinguished for Cu_0.04_Zn_0.96_O NPs, suggesting that the substitution of Zn by Cu in the ZnO wurtzite structure was successful. This may have been due to the small differences between the Zn and Cu ionic radii and their similar electronegativities [37]. Indeed, Cu^2+^ and Zn^2+^ ions have electronegativity values of 1.91 and 1.65 [38], and ionic radii of 0.73 Å and 0.74 Å, respectively [39]. The morphology of the Cu_0.04_Zn_0.96_O NPs was studied using TEM. As shown in Figure 2c,d, the sample consisted of triangular NPs with a quite narrow size distribution in the range of 40–56 nm [40].

**Figure 3:**
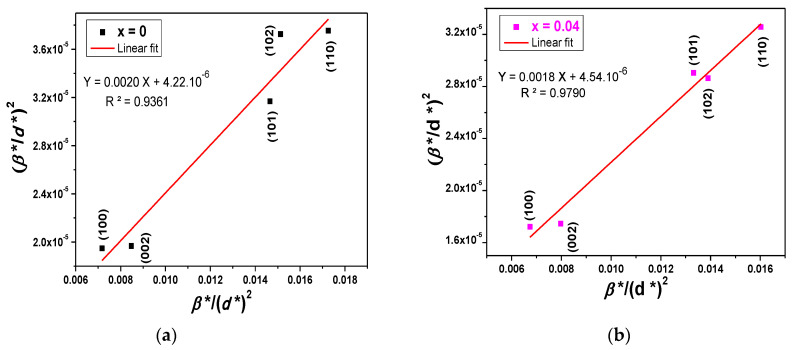
Halder–Wagner plots of Cu*_x_*Zn_1−*x*_O NPs (*x* = 0 and 0.04).

The average crystallite size and strain of the Cu*_x_*Zn_1−*x*_O NPs were extracted by fitting the Halder–Wagner (H–W) plots, as shown in Figure 3a,b. The obtained average crystallite size, lattice parameters, dislocation density, and strain rate for all samples are given in Table 1. As can be seen, the average size increased with the Cu concentration, which proved that the incorporation of Cu^2+^ in the host ZnO lattice enhanced the growth rate of NPs [41]. It also induced a slight decrease in the lattice parameters due to the small difference in the ionic radius between Zn and Cu [21].

**Figure 4:**
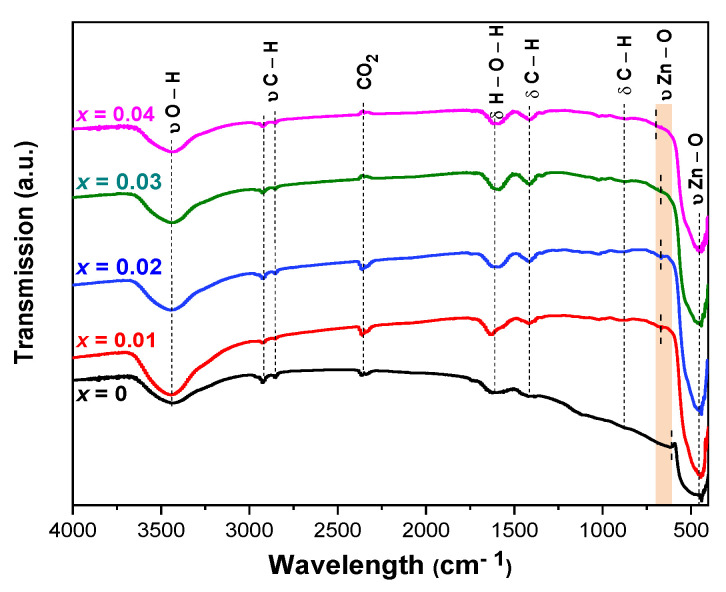
Fourier-transform infrared (FTIR) spectra of Cu*_x_*Zn_1−*x*_O NPs (*x* = 0, 0.01, 0.02, 0.03, and 0.04).

FTIR was used to gain information about the chemical bonding and to identify the elemental constituents of our samples [42]. The FTIR spectra for undoped and Cu-doped ZnO NPs are presented in Figure 4. The broad absorption band at 3434 cm^−1^ was assigned to the stretching vibration of hydroxyl groups. These vibrations came from water molecules absorbed onto the Cu*_x_*Zn_1−*x*_O NPs. The band at 1606 cm^−1^ was assigned to the bending mode of H–O–H [43]. The series of peaks at 1423, 2856, and 2922 cm^−1^ were due to the C–H bending and stretching vibrations of alkane chains (most likely from the zinc acetate precursor), and the peak around 867 cm^−1^ corresponded to the C–H bending vibration [44,45]. The peaks at about 2351 cm^−1^ were due to CO_2_ molecules present in the air [46]. The intense absorption band at about 450 cm^−1^ and relatively weak absorption peak at 621 cm^−1^ in the FTIR spectra corresponded to the characteristic stretching vibrations of Zn–O and confirmed the formation of ZnO [47]. As the copper content increased in the ZnO lattice, its structure changed and presumably led to changes in the shape of low wavenumber features. The stretching frequency at 621 cm^−1^ for undoped ZnO was noticeably shifted to a higher frequency up to 696 cm^−1^ for *x* = 0.04. This agrees with the fact that copper atoms are lighter than Zn atoms, resulting in an upward shift of the fundamental transverse optical phonon mode [19].

**Figure 5:**
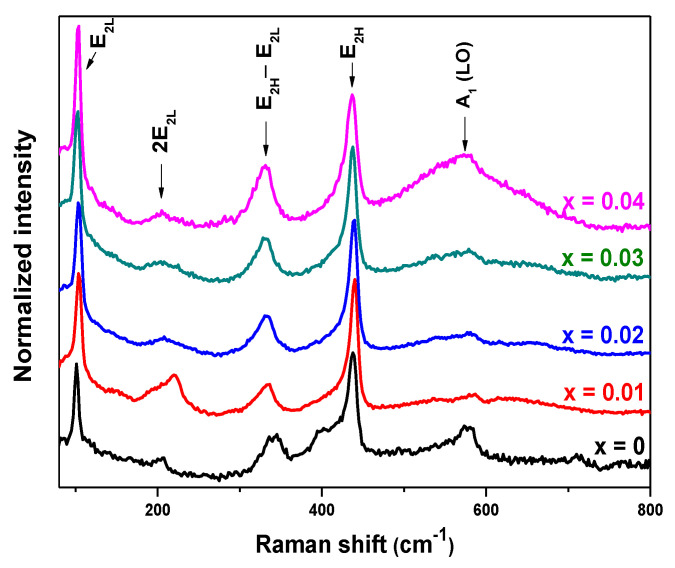
Raman spectra of Cu*_x_*Zn_1−*x*_O NPs (*x* = 0, 0.01, 0.02, 0.03, and 0.04). LO: longitudinal optical.

Raman spectroscopy is a powerful diagnostic tool that is used to obtain information about the crystallization, structural disorder, and defects in the micro- and nanostructures [48]. The Raman spectra of the Cu*_x_*Zn_1−*x*_O NPs are shown in Figure 5. Peaks located at about 101, 437, and 584 cm^−1^ were assigned to the E_2L_, E_2H_, and A_1_ longitudinal optical (LO) phonons of hexagonal ZnO. The Raman peak positioned at about 203 cm^−1^ corresponded to the 2E_2L_ second-order scattering. Additionally, the other Raman peak at about 333 cm^−1^ was attributed to the E_2H_−E_2L_ multiphonon scattering [49,50,51]. The E_2H_ mode is mainly caused by the vibration of oxygen atoms, which are sensitive to internal stress [52]. It is characteristic of the hexagonal wurtzite structure of ZnO nanostructures [53]. The E_2H_ Raman line broadens with increasing Cu content [52,54]. Doping introduces chemical disorder (to which Raman spectroscopy is sensitive) and can also introduce lattice defects and disorder. Defect-induced Raman modes manifest in defective crystals because the Raman selection rules are relaxed [55]. As the copper concentration increased, the A_1_ (LO) peak broadened and shifted about 13 cm^−1^ toward lower wavenumbers. This result is consistent with already reported work [56]. The shift and broadening of the A_1_ (LO) phonon peak were assigned to the scattering contributions of the A_1_ (LO) branch outside the Brillouin zone center [57]. Light scattering by the A_1_ (LO) phonon is commonly attributed to oxygen vacancies or zinc interstitials in ZnO [58].

**Figure 6:**
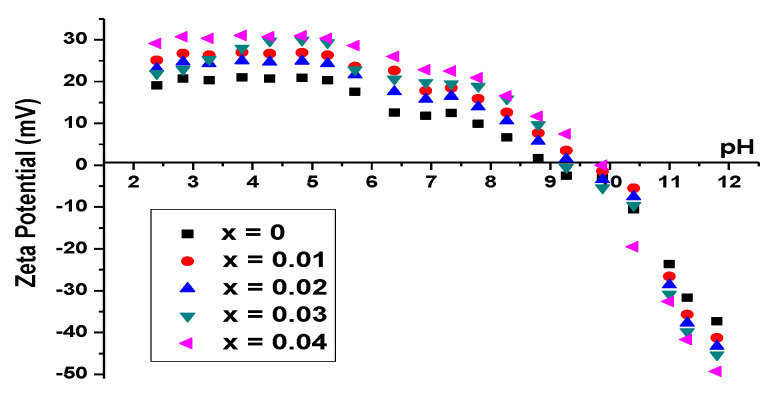
Zeta potential curves of Cu*_x_*Zn_1−*x*_O NPs (*x* = 0, 0.01, 0.02, 0.03, and 0.04) in 10^−2^ M NaCl.

The zeta potential measurements of the Cu*_x_*Zn_1−*x*_O NPs (*x* = 0, 0.01, 0.02, 0.03, and 0.04) are shown in Figure 6. The incorporation of Cu into the ZnO increased the electrical charge of the NPs and caused a shift of the IEP toward higher values. ZnO has an isoelectric point around pH 9, which increased to 9.9 for Cu_0.04_Zn_0.96_O NPs, suggesting that the surface-charging behavior was changed as the Cu content increased. This proved that the incorporation of Cu enhanced the adsorption of hydroxyl on the NPs’ surfaces and hence increased the affinity to protons, which resulted in the increase of the IEP [59]. Above the IEP, the NPs carried a net negative charge and thus a high affinity for positively charged species.

### 2.2. Electrochemical Characterization of the Cu_x_Zn_1−x_O/ITO Modified Electrodes

**Figure 7:**
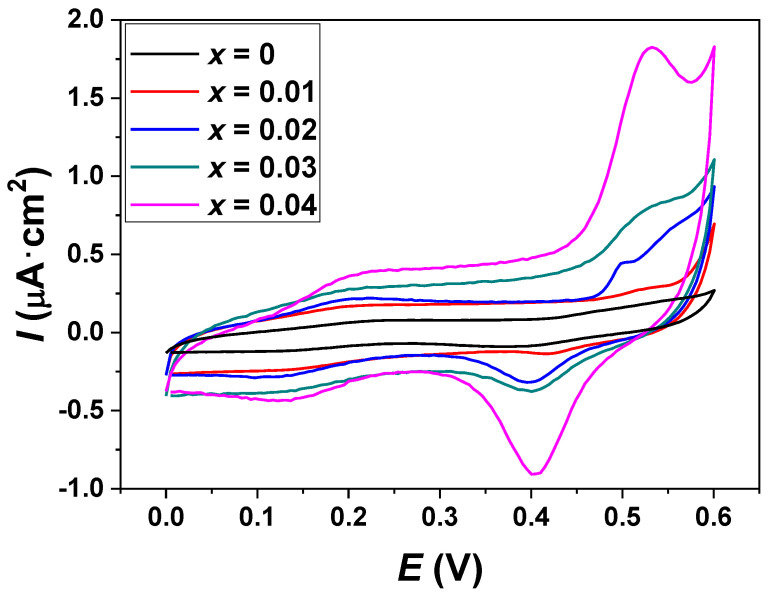
Cyclic voltammograms of the Cu*_x_*Zn_1−*x*_O/ITO modified electrodes that were obtained in the absence of glucose at a scan rate of 50 mV·s^−1^.

The cyclic voltammograms of doped and undoped ZnO samples in 0.1 M NaOH are shown in Figure 7. At the ZnO/ITO electrode, a low oxidation current existed. Doped ZnO samples showed an important Cu(II)–Cu(III) redox peak, which enhanced and shifted toward a lower potential as the Cu content increased. These enhanced peak current and potential shift were attributed to the improved conductivity and electrocatalytic properties due to the copper doping [60]. Interestingly, the electrocatalytic activity of the Cu_0.04_Zn_0.96_O/ITO electrode was noticeably different from the others. It showed a couple of strong and well-defined redox peaks. The increased current density occurred because of the charge carriers approaching the conduction band of ZnO, which could be involved in the charge transfer at the metal oxide/electrolyte interface [61]. From this observation, the increase in dopant concentration provided a higher electrical conductivity, faster electron transfer kinetics, and excellent electroactive surface area, thereby improving the electrochemical response [62].

**Figure 8:**
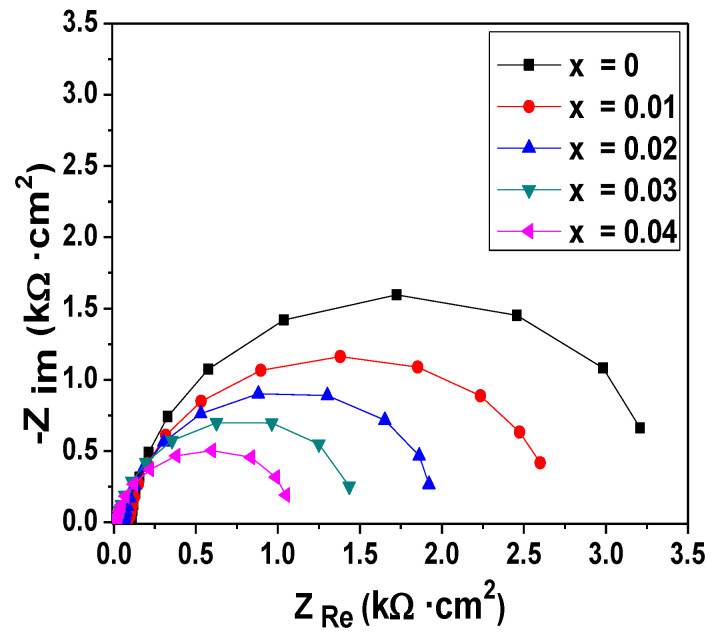
Nyquist plots for different electrodes in 0.1 M NaOH that were obtained in the absence of glucose.

Electrochemical impedance spectroscopy (EIS) is a particularly important tool for investigating the interfacial properties of the modified electrodes. This method has recently attracted extensive attention in the field of analytical sciences since it can provide complementary information about the reaction dynamics and the membrane/solution structure. Figure 8 shows the Nyquist plots of the EIS for Cu*_x_*Zn_1−*x*_O/ITO (*x* = 0, 0.01, 0.02, 0.03, and 0.04) electrodes in 0.1 M NaOH. We observed that the semicircle diameter of the sensors decreased dramatically with increasing *x* values. This decrease was attributed to the variation of the electric parameters of the membrane with increasing Cu doping content.

**Figure 9:**
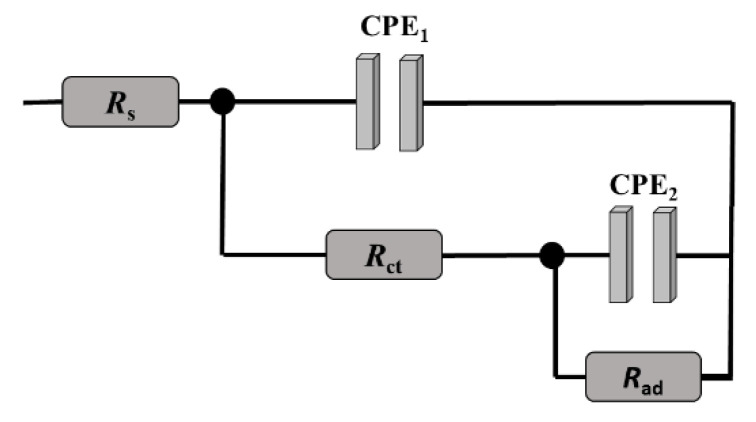
Equivalent electrical circuit that was used to model the impedance spectra.

The EIS data were fitted using ZView software (Southern Pines, NC, USA) to determine these parameters. An example of the Nyquist and Bode diagrams’ fits of the Cu_0.04_Zn_0.96_O/ITO electrode is presented in the Supplementary Information (Figure S2). This figure shows that the phase plot presented a large phase angle maximum, which proved that the two-phase angle maxima merged toward each other. Hence, the Nyquist plots were modeled as an overlapping of two semicircles. The equivalent circuit that best fit the Nyquist plots with a low total error is presented in Figure 9. It was composed of the electrolyte resistance (*R*_s_) in series with two parallel combinations of a resistance and a constant phase element. The first one refers to the high-frequency loop. The electrochemical phenomenon occurring at the ITO/electrolyte interface was modeled using the charge transfer resistance (*R*_ct_) and the constant phase element CPE_1_. The second loop (*R*_ad_, CPE_2_) was introduced to describe the adsorption on the electrode surface in the low-frequency region. The CPE element is a non-ideal capacitor that can be expressed using the following equation [63]: $\;\mathrm{CPE}=1/Q{\left(j\omega \right)}^{n}$, where *Q* is a frequency-independent term that refers to the homogeneity, roughness, and surface porosity, *j* is an imaginary number (*j* = $\sqrt{-1}$), *ω* is the angular frequency (*ω* = 2π*f*), and 0 < *n* < 1. CPE becomes more capacitive when the value of *n* tends to 1 [64]. The fitted values, along with the total error (*χ*^2^), are given in Table 2. When increasing the Cu doping, there was an increase in the CPE values, denoting the accumulation of charge at the electrode surface, which was accompanied by a gradual decrease in *R*_ct_ and *R*_ad_. The decrease in *R*_ct_ revealed the enhancement of the electrical conductivity. The dramatic decrease in *R*_ad_ resulted from the improved adsorption ability with increasing the Cu doping because it mainly took place at the Cu electrocatalytic active sites. Hence, Cu doping provided many active sites and high surface energies. The lowest resistances were obtained at the Cu_0.04_Zn_0.96_O/ITO electrode. This is in agreement with the cyclic voltammetry results and confirms that Cu_0.04_Zn_0.96_O/ITO electrodes are good candidates for the electrochemical sensing of glucose.

### 2.3. Electrochemical Study of Glucose Sensing at the Cu_0.04_Zn_0.96_O/ITO Electrode

#### 2.3.1. Cyclic Voltammetry (CV)

**Figure 10:**
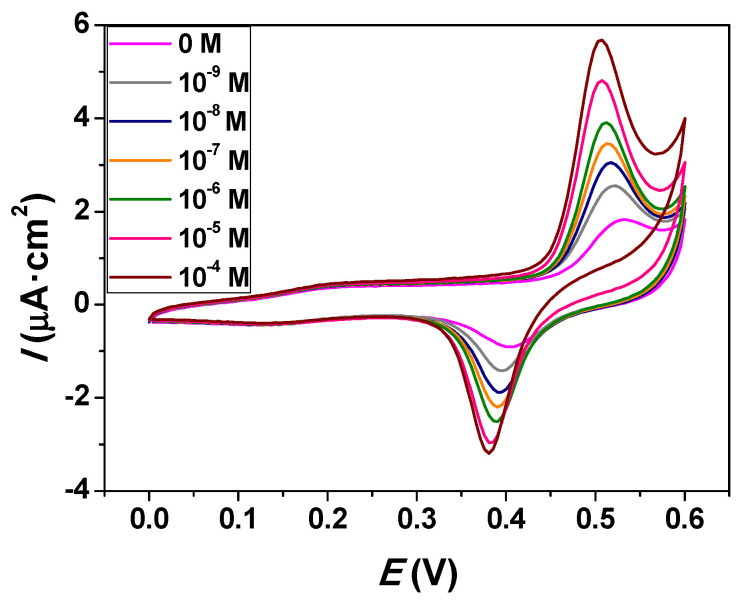
Cyclic voltammograms of the Cu_0.04_Zn_0.96_O/ITO electrode in 0.1 M NaOH containing various concentrations of glucose.

CV was used to study the effect of the glucose concentration on the electrocatalytic performance of the Cu_0.04_Zn_0.96_O/ITO electrode in 0.1 M NaOH. Figure 10 represents the cyclic voltammograms of the Cu_0.04_Zn_0.96_O/ITO electrode for various concentrations of glucose at a scan rate of 50 mV·s^−1^. A well-defined pair of redox peaks was observed at 0.53 V and 0.40 V. These peaks were attributed to the Cu(II)/Cu(III) redox couple in the alkaline medium. However, the anodic and cathodic peak currents increased with increasing glucose concentration, proving that glucose was oxidized by the Cu_0.04_Zn_0.96_O/ITO electrode. The cyclic voltammograms of Cu_0.05_Zn_0.95_O NPs in the absence and presence of glucose are presented in the Supplementary Information (Figure S3). According to the cyclic voltammograms, the Cu_0.04_Zn_0.96_O NPs were more promising for detecting glucose due to the presence of sharper redox peaks than the Cu_0.05_Zn_0.95_O NPs. It seems that the appearance of the secondary CuO phase with increasing Cu doping up to *x* = 0.05 affected the NPs’ ability to detect glucose.

**Figure 11:**
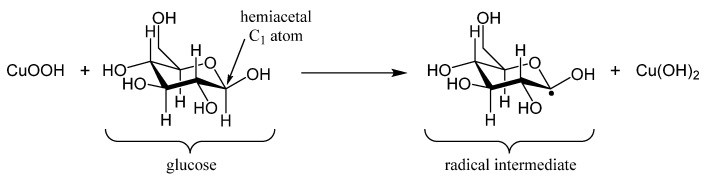
Dehydrogenation of the glucose molecule at the hemiacetalic carbon 1 atom (C1) to form an adsorbed radical intermediate.

**Figure 12:**
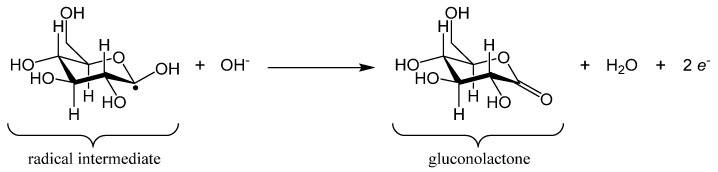
The radical intermediate reacted quickly with hydroxyl anions in the electrolyte to form gluconolactone.

During the electrocatalytic oxidation of glucose, Cu(0) was first oxidized into Cu(II) (reaction 1 below). Cu(II) species were then oxidized into Cu(III) (reaction 2). Then, the oxidation of glucose was mainly based on the Cu(OH)_2_/CuOOH redox couple (reaction 3). Therefore, the loss of electrons at the surface to produce the Cu(III) species instantly activated the electrode and acted as a strong oxidant, with empty d-orbitals being available to adsorb glucose. The glucose electro-oxidation process might have consisted of the adsorption of a glucose molecule on the electrode surface via dehydrogenation of the glucose molecule at the hemiacetalic carbon 1 atom (C_1_) to form an adsorbed radical intermediate (Figure 11), which in turn reacted quickly with hydroxyl anions in the electrolyte to form gluconolactone (Figure 12). Finally, the resulting gluconolactone spontaneously converted into gluconic acid via hydrolysis (reaction 4). The electrocatalytic oxidation reactions can be written as follows [35]:

(1) $$\mathrm{Cu}+2\;{\mathrm{OH}}^{-}\to \mathrm{Cu}{\left(\mathrm{OH}\right)}_{2}\;+2\;e^{-},\;\;$$

(2) $$\mathrm{Cu}{\left(\mathrm{OH}\right)}_{2}+{\mathrm{OH}}^{-}\to \mathrm{CuOOH}+H_{2}O+e^{-},$$

(3) $$\mathrm{CuOOH}+\mathrm{glucose}\to \mathrm{Cu}{\left(\mathrm{OH}\right)}_{2}+\mathrm{gluconolactone},$$

(4) $$\mathrm{gluconolactone}\overset{\mathrm{hydrolysis}}{\to }\mathrm{gluconic}\;\mathrm{acid}.$$

#### 2.3.2. Electrochemical Impedance Spectroscopy

**Figure 13:**
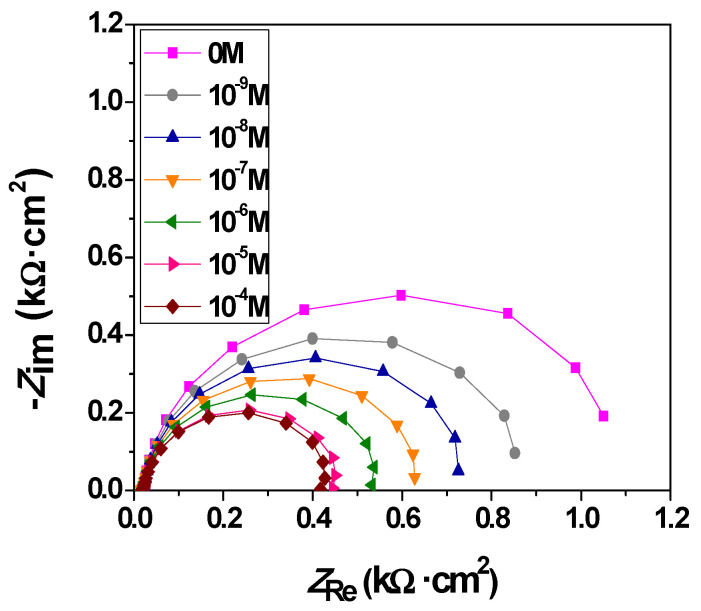
Nyquist plots for the Cu_0.04_Zn_0.96_O/ITO electrode in 0.1 M NaOH solution for 0–10^−4^ M glucose concentration ranges.

Nyquist plots for the Cu_0.04_Zn_0.96_O/ITO electrode in 0.1 M NaOH solution for different glucose concentrations, ranging from 1 nM to 100 µM, are shown in Figure 13. They consisted of the overlapping of two semicircles whose diameters decreased with increasing glucose concentrations. This decrease proved the good interaction between glucose and the sensing membrane. The electrochemical properties of the membrane were quantified by fitting the impedance plots using the circuit already presented in Figure 9. The values of the electrical parameters that were obtained by fitting the data are given in Table 3. The solution resistance (*R*_s_) was almost constant for all the concentrations of glucose. The CPE values were not affected by the increase in glucose concentration, whereas *R*_ct_ and *R*_ad_ decreased significantly for increasing glucose concentrations. On the one hand, the decrease in *R*_ct_ revealed an enhancement in the electrical conductivity that assisted the needed conductive pathways by providing a high electron transfer between the sensing membrane and the glucose. On the other hand, the decrease in *R*_ad_ was due to the large surface area, which in turn improved the availability of metallic centers that facilitated the interaction with glucose and led to their accessibility to an active site. Thereby, this result revealed the high electrocatalytic activity of the Cu_0.04_Zn_0.96_O/ITO electrode toward glucose oxidation as it improved the conversion of glucose into gluconolactone via the oxidation of Cu(OH)_2_ to CuOOH.

#### 2.3.3. Differential Pulse Voltammetry (DPV) Detection of Glucose

**Figure 14:**
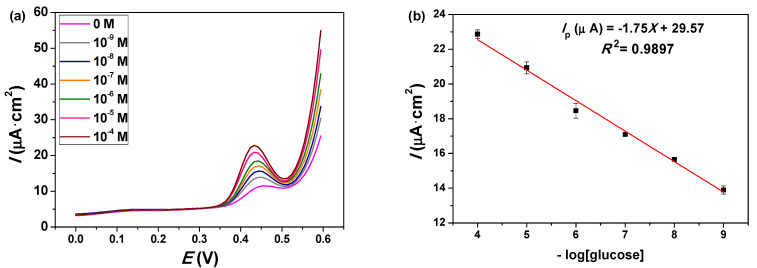
(**a**) Differential pulse voltammograms of the Cu_0.04_Zn_0.96_O/ITO electrode for different glucose concentrations. (**b**) Linear calibration curve of the peak currents vs. the logarithmic glucose concentration.

Figure 14a shows the DPV analysis of the Cu_0.04_Zn_0.96_O/ITO electrode for different glucose concentrations. Well-defined oxidation peaks of glucose were observed. The calibration plot (Figure 14b) shows that the DPV peak current was proportional to the logarithmic concentration of glucose in the 1 nM to 100 μM range. The equation of the calibration plot was *I*_p_ (μA) = −1.75*X* + 29.57 (*R*^2^ = 0.9897). The limit of detection (LOD) was estimated to be 0.7 nM based on LOD=3×SD/S, where *SD* is the standard deviation of the response and *S* is the slope of the calibration curve [65]. A comparison of the analytical performance of the Cu_0.04_Zn_0.96_O/ITO electrode with other electrochemical sensors for glucose detection is shown in Table 4. The low limit of detection was associated with the high specific surface ratio of the NPs and the improved interaction between the modified electrode surface and analytes. Therefore, our Cu_0.04_Zn_0.96_O/ITO-modified electrode showed good analytical properties that were suitable for the quantification of glucose down to nanomolar concentrations.

### 2.4. Interferences and Practical Application of Cu_0.04_Zn_0.96_O/ITO Electrodes

**Figure 15:**
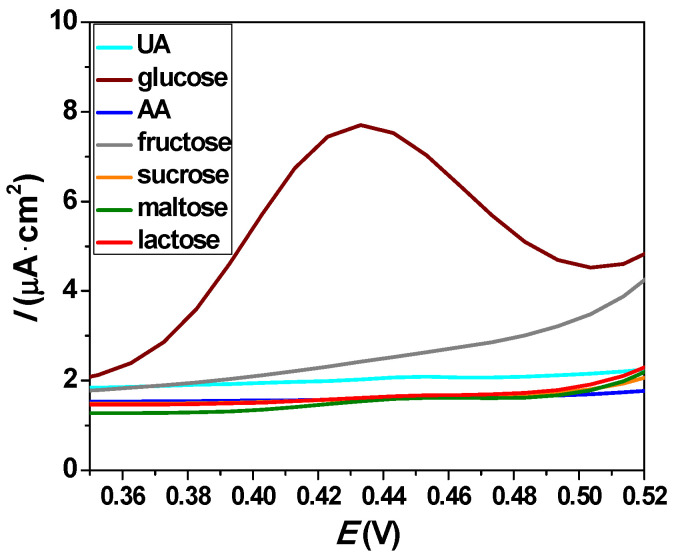
Differential pulse voltammetry of the Cu_0.04_Zn_0.96_O/ITO electrode in the presence of a 10^−4^ M concentration of uric acid (UA), glucose, ascorbic acid (AA), fructose, sucrose, maltose, and lactose in a 0.1 M NaOH solution.

A challenge regarding non-enzymatic glucose sensors is the prevention of interfering reagents. We examined the specificity of the Cu_0.04_Zn_0.96_O/ITO glucose sensor toward interfering species, such as ascorbic acid (AA), uric acid (UA), fructose, sucrose, maltose, and lactose. Figure 15 shows the DPV of the Cu_0.04_Zn_0.96_O/ITO electrode in the presence of glucose and the above-mentioned species. The results indicated that the response of our electrode for these molecules was negligible compared to the response to glucose [74]. Therefore, the presence of these interfering species had no influence on the detection of glucose. The developed sensor based on Cu_0.04_Zn_0.96_O NPs was highly specific toward the sensing of glucose.

### 2.5. Practical Applications

The practical feasibility of the developed sensor electrode was confirmed by the detection of glucose in human blood serum samples. The concentration of glucose was measured via the addition of fresh serum that was diluted ten times into a 0.1 M NaOH solution. The recoveries were evaluated in the standard addition way by adding a glucose concentration to the resulting solution. Analytical recoveries of the added glucose into the human serum samples were between 80 and 99.6% (Table 5). These recoveries of glucose proved the potential of our Cu_0.04_Zn_0.96_O/ITO electrode to be used in practical applications.

## 3. Materials and Methods

### 3.1. Chemicals and Reagents

Zinc acetate dihydrate (Zn(CH_3_COO)_2_·2H_2_O, 99.99% purity), copper chloride dihydrate (CuCl_2_·2H_2_O), polyvinyl alcohol Mowiol 4–88 (88% hydrolysis/MW: 31 kDa), sodium hydroxide (NaOH pellets, ≥97.0%), methanol, D-(+)-glucose, ascorbic acid (AA), uric acid (UA), fructose, sucrose, maltose, and lactose were purchased from Sigma-Aldrich (St. Louis, MO, USA). They were of analytical grade and used as received, without further purification. Distilled water was used for the preparation of all aqueous solutions.

### 3.2. Synthesis of Cu_x_Zn_1−x_O NPs

The Cu*_x_*Zn_1−*x*_O (*x* = 0, 0.01, 0.02, 0.03, and 0.04) NPs were synthesized using a simple and low-cost sol-gel method. Briefly, 2 g of zinc acetate was solubilized in 14 mL methanol under magnetic stirring at 25 °C for 2 h. Copper was added according to the desired [Cu]/([Zn] + [Cu]) ratio (0, 0.01, 0.02, 0.03, and 0.04) under magnetic stirring until complete dissolution of the precursors was achieved. Then, the suspensions were centrifuged at 1512 RCF (relative centrifugal force) for 20 min and washed with distilled water and methanol. The wet powders were then placed in an autoclave and dried at 45 °C and 7.2 MPa in the supercritical condition of ethyl alcohol (EtOH) for 2 h [75].

### 3.3. Electrode Preparation

The NPs were dispersed in distilled water and added to an aqueous polyvinyl alcohol (PVA) solution. The addition of PVA facilitated the dispersion of NPs in water and their immobilization on the substrate. The resulting PVA–Cu*_x_*Zn_1−*x*_O suspensions were then kept under magnetic stirring for 10 min. The obtained suspensions were spin-coated on indium-doped tin oxide (ITO) substrates. The spinning time was 30 s and the spinning rate was 56 RCF. Finally, the electrodes were dried in an oven at 120 °C for 1 h.

### 3.4. Characterization of the Cu_x_Zn_1−x_O NPs

The crystalline structure of the Cu*_x_*Zn_1−*x*_O (*x* = 0, 0.01, 0.02, 0.03, and 0.04) NPs was studied using XRD with a Bruker AXS D8 Advance X-ray diffractometer (Billerica, MA, USA) using the CuKα radiation (wavelength λ = 1.5418 Å). The diffraction peaks were broadened by the crystalline size, the intrinsic strains, and instrumental broadening. Crystalline silicon was used as a standard reference specimen to decouple the instrumental contributions. The instrument-corrected broadening *β* corresponding to each diffraction peak of the Cu*_x_*Zn_1−*x*_O NPs was determined according to Gaussian profile (5) [76].

(5) $$\beta^{2}=\beta_{m}^{2}-\beta_{i}^{2},$$

where *β* is the corrected broadening, *β_m_* is the measured broadening, and *β_i_* is the instrumental broadening. Then, the corrected physical broadening was used to estimate the crystallite size of Cu*_x_*Zn_1−*x*_O NPs using the H–W method [77]. This method considers both the size and strain effects on the XRD peak broadening and is relevant in the case of Gaussian and Voigt profiles [78]. The crystallite size and strain of the Cu*_x_*Zn_1−*x*_O NPs were estimated using the H–W equation (Equation (6)) [79]:

(6) $${\left(\beta^{*}/d^{*}\right)}^{2}=\frac{1\;}{\varepsilon }\left(\beta^{*}/{\left(d^{*}\right)}^{2}\right)+{\left(\eta /2\right)}^{2},$$

where *ε* is the parameter related to the crystallite size, *η* is the parameter linked to strain, and $\beta^{*}=\beta \cos\theta /\lambda$ and $d^{*}=2\sin\theta /\lambda$ are the reduced coordinates as a function of the diffraction angle *θ* and the wavelength *λ*. The H–W plots for Cu*_x_*Zn_1−*x*_O NPs were obtained by plotting ${\left(\beta^{*}/d^{*}\right)}^{2}$ versus $\beta^{*}/{\left(d^{*}\right)}^{2}$. The crystalline size, *D*, was obtained from the slope of the fit and the microstrain from the intercept. Furthermore, the lattice parameters *a* and *c* of the Cu*_x_*Zn_1−*x*_O NPs were estimated according to the following Equation (7) [80]:

(7) $$a=\lambda /\sqrt{3}\sin\theta \;\mathrm{and}\;c=\lambda /\sin\theta .$$

In addition, the dislocation density *δ* of the NPs was calculated using Williamson and Smallman’s equation (Equation (8)):

(8) $$\delta =n/D^{2},$$

where n is a constant that is usually close to one [81].

The morphology and the structure of the synthesized NPs were investigated using HR-TEM with a JEOL JEM 2100F microscope (TEM, Tokyo, Japan) operating at 200 kV (point-to-point resolution of 0.19 nm). The samples were prepared by evaporating a diluted suspension of NPs in deionized (DI) water on a carbon-coated copper grid. About 150 NPs were counted to estimate the average size (Image J software, 1.52a, NIH, MD, USA).

FTIR measurements were recorded on a Bruker IFS 28 (Billerica, MA, USA) using the OPUS software (version 3.1) in the 4000–400 cm^−1^ wavenumber range, with a resolution of 4 cm^−1^ and a total of 10 scans per measurement. Pellets were made with 1 mg of sample mixed with 150 mg of dried KBr.

Raman spectra were recorded on a Renishaw inVia microspectrometer (Wotton-under-Edge, UK) with the 532 nm excitation line of a doubled Nd^3+^ YAG laser. The laser beam was focused onto a 10 μm^2^ area and the power was kept below 1 mW to avoid sample heating. The raw spectra were baseline-corrected.

Zeta potentials were measured with a Malvern Zetasizer Nano ZSP instrument (Worcestershire, UK). The suspensions of NPs were prepared in 10^−2^ M NaCl aqueous solutions. The pH of the suspension was adjusted from 2 to 12 by adding HCl (0.1 M) or NaOH (0.1 M and 0.01 M) solutions.

### 3.5. Electrochemical Characterizations

The electrochemical measurements were performed using a Metrohm Potentiostat Autolab PGSTAT 20 (ECO CHEMIE Ultrecht, The Netherlands) running the Frequency Response Analysis (FRA) and General Purpose Electrochemical System (GPES) software (North Carolina, USA). The measurements were carried out in an electrochemical cell involving a conventional three-electrode system that was equipped with a platinum plate as a counter-electrode, a saturated calomel electrode as the reference electrode, and a sensitive membrane based on Cu*_x_*Zn_1−*x*_O NPs as the working electrode. All experiments were carried out at 25 °C using 0.1 M NaOH as the aqueous electrolyte, which was usually selected for non-enzymatic glucose detection [72,82]. This alkaline medium facilitated the formation of higher oxidized species, such as Cu(OH)_2_ and CuOOH, which significantly affected the glucose electro-oxidation. Cyclic voltammetry measurements were done with a scan rate of 50 mV·s^−1^ by cycling the potential from 0 to 0.6 V. Electrochemical impedance spectroscopy was performed with a sinusoidal excitation signal with a 10 mV amplitude and the impedance was recorded in the frequency range from 100 to 0.1 Hz. The optimal working potential was adjusted to 0.7 V (the optimization of the polarization potential is described in supporting information section (Supplementary Materials Figure S3)). The experimental data of the EIS Nyquist plots were fitted with the ZView software. Differential pulse voltammetry peak currents were recorded between 0 and 0.6 V at a scan rate of 50 mV·s^−1^. For the glucose detection, multiple solutions of different glucose concentrations were made by following serial dilutions from an initial stock concentration of 10^−2^ M. The glucose solutions were tested in increasing concentrations, where the first one was made from the prepared solution of 10^−6^ M, which led to a final concentration of 10^−9^ M in the electrochemical cell.

## 4. Conclusions

In summary, the influence of Cu doping on the physicochemical properties of ZnO NPs was successfully studied using XRD, TEM, FTIR and Raman spectroscopies, and zeta potential measurements. The use of Cu*_x_*Zn_1−*x*_O/ITO (*x* = 0, 0.01, 0.02, 0.03, and 0.04) as an electrode material for non-enzymatic glucose sensors was successfully demonstrated. EIS, CV, and DPV analyses of the Cu*_x_*Zn_1−*x*_O/ITO modified electrodes showed that copper doping significantly improved the electrochemical properties. Owing to the large surface area and strong adsorption capability, the Cu_0.04_Zn_0.96_O/ITO sensor exhibited an excellent electrocatalytic activity toward glucose in an alkaline medium (0.1 M NaOH). It exhibited high sensitivity, a low limit of detection, and quantification of glucose over a wide concentration range. Moreover, the selectivity was excellent, as shown by the good resistance to interferences from some common interfering species. The Cu_0.04_Zn_0.96_O/ITO sensing electrode was also suitable for the determination of glucose in human serum samples. This simple, low-cost, and environmentally friendly sensor with excellent detection properties can potentially pave the way for the commercial production of a glucose sensor.

## Figures and Tables

**Table 1:** Data for the value of the average crystallite size, lattice parameters (*a* and *c*), strain, and dislocation density (*δ*) of Cu*_x_*Zn_1−*x*_O NPs.

NPs	Lattice Constants (nm)		Strain	*δ* (10^−4^) (nm^−2^)	Particle Size (nm)
	*a*	*c*			
ZnO	0.2862	0.4957	0.1	3.95	50.3
Cu_0.01_Zn_0.99_O	0.2861	0.4956	0.09	3.92	50.5
Cu_0.02_Zn_0.98_O	0.2860	0.4955	0.08	3.84	51
Cu_0.03_Zn_0.97_O	0.2859	0.4953	0.08	3.81	51.2
Cu_0.04_Zn_0.96_O	0.2856	0.4947	0.08	3.11	56.7

**Table 2:** Electrical parameter values that were obtained from fitting the impedance spectra of different electrodes.

Electrodes	*R*_s_ (10^−3^) (kΩ·cm^2^)	CPE_1_ (µF)	*n*_1_	*R*_ct_ (10^−3^) (kΩ·cm^2^)	CPE_2_ (µF)	*n*_2_	*R*_ad_ (10^−3^) (kΩ·cm^2^)	*χ*^2^ (10^−4^)
ZnO	66.4	7.2 ± 0.1	0.98	706.9 ± 0.2	2.6 ± 0.1	0.98	2520.3 ± 0.2	5.56
Cu_0.01_Zn_0.99_O	64.8	8.6 ± 0.2	0.99	636.2 ± 0.2	6.4 ± 0.1	0.99	2028.6 ± 0.1	7.26
Cu_0.02_Zn_0.98_O	47.9	19.1 ± 0.1	0.98	569.3 ± 0.1	9.9 ± 0.1	0.97	1327.8 ± 0.1	5.12
Cu_0.03_Zn_0.97_O	25.2	34.5 ± 0.1	0.97	542.6 ± 0.1	13.9 ± 0.1	0.97	990.3 ± 0.2	2.89
Cu_0.04_Zn_0.96_O	17.5	35.6 ± 0.2	0.99	302.9 ± 0.1	15.7 ± 0.2	0.99	810.5 ± 0.1	3.74

**Table 3:** Fitting data of the Cu_0.04_Zn_0.96_O/ITO sensing electrode for different concentrations of glucose.

Glucose Concentration (M)	*R*_s_ (10^−3^) (kΩ·cm^2^)	CPE_1_ (µF)	*n*_1_	*R*_ct_ (10^−3^) (kΩ·cm^2^)	CPE_2_ (µF)	*n*_2_	*R*_ad_ (10^−3^) (kΩ·cm^2^)	*χ*^2^ (10^−4^)
0	17.5	35.6 ± 0.2	0.99	302.9 ± 0.1	15.7 ± 0.2	0.99	810.5 ± 0.1	3.74
10^−9^	17.4	36.6 ± 0.5	0.98	243.2 ± 0.1	16.1 ± 0.2	0.99	607.2 ± 0.1	5.62
10^−8^	17.2	36.4 ± 0.6	0.99	192.2 ± 0.1	15.8 ± 0.1	0.99	508.4 ± 0.2	2.35
10^−7^	17.5	36.2 ± 0.2	0.97	151.2 ± 0.2	15.6 ± 0.3	0.98	450.7 ± 0.2	6.24
10^−6^	17.0	36.1 ± 0.1	0.98	131.8 ± 0.1	15.2 ± 0.1	0.99	390.3 ± 0.1	6.45
10^−5^	16.8	35.9 ± 0.3	0.97	102.1 ± 0.2	16.2 ± 0.2	0.98	342.5 ± 0.1	3.59
10^−4^	16.7	36.3 ± 0.5	0.98	94.2 ± 0.2	16.2 ± 0.1	0.99	326.6 ± 0.1	6.12

**Table 4:** Comparison of the sensing performance found in this work with similar non-enzymatic glucose sensors.

Electrode Material	Linear Range (μM)	Detection Limit (μM)	Analytical Technique	Reference
Ni(II)-CP/C_60_	10 to 3 × 10^3^	4.3	Amp	[66]
Mucilage-AgNPs/GC	10 to 2.2 × 10^3^	10	SWV	[67]
Net-Co_3_(PO_4_)_2_/NG	0.05 to 2 × 10^3^	1	Amp	[68]
ZnO/CeO_2_ whisker	0.5 to 300	0.22	DPV	[69]
Au@Cu_2_O	50 to 2 × 10^3^	18	CV	[70]
HLTH/NF	15 to 8 × 10^3^	1.49	Amp	[71]
Au-TiO_2_ NTs	50 to 3 × 10^3^	50	CV	[72]
CuO nanodisks	2 to 2.5 × 10^3^	0.2	Amp	[73]
Cu_0.04_Zn_0.96_O	10^−3^ to 100	7 × 10^−4^	DPV	This work

Ni(II)-CP/C60: Ni(II)-coordination polymer and fullerene (C60), Amp: amperometry, AgNPs: silver NPs, GC: glassy carbon, SWV: squarewave voltammetry, Net-Co_3_(PO_4_)_2_/NG: networked cobaltous phosphate decorated with nitrogen-doped reduced graphene oxide, DPV: differential pulse voltammetry, CeO_2_: cerium oxide, Cu_2_O: cuprous oxide, AuNPs: gold NPs, HLTH/NF: hollow triple-layered hydroxide modified nickel foam, Au-TiO_2_NTs: gold layers deposited onto TiO_2_ nanotubes, CuO: copper oxide.

**Table 5:** Determination of glucose in human serum samples by the Cu_0.04_ Zn_0.96_O/ITO electrode.

Sample	Added Concentration (μM)	Found (μM)	Recoveries (%)
Human serum	0.05	0.04	80.0
	5	4.98	99.6
	500	497	99.4

## Data Availability

The datasets used and/or analyzed during the current study are available from the corresponding authors on reasonable request.

## Supplementary Materials

The following are available online. Figure S1: X-ray diffraction analysis of Cu_0.05_Zn_0.95_O NPs, Figure S2: Example of the fits of the Nyquist and Bode diagrams of Cu_0.04_Zn_0.96_O/ITO, Figure S3: Cyclic voltammograms of Cu_0.05_Zn_0.95_O NPs in the absence and presence of glucose, Figure S4: Optimization of the polarization potential for the EIS measurements.

## Sample Availability

Samples of the compounds are available from the authors.
