# Supplementary material for: Cu-Doped ZnO Nanoparticles for Non-Enzymatic Glucose Sensing
Source: Molecules. 2021 Feb 10;26(4):929. doi: 10.3390/molecules26040929 (PMC7916517; doi:10.3390/molecules26040929)
Supplement: Supplementary file 1 [file molecules-26-00929-s001.pdf]

Supplementary Information

Cu-Doped ZnO Nanoparticles for Non-enzymatic Glucose Sensing

Amira Mahmoud [1,2], Mosaab Echabaane [3], Karim Omri [4], Julien Boudon [2], Lucien Saviot [2], Nadine Millot [2,*] and Rafik Ben Chaabane [1]

[1] Laboratory Interfaces and Advanced Materials (LIMA), Faculty of Science of Monastir, University of Monastir, 5019 Monastir, Tunisia; Amira_Mahmoud@etu.u-bourgogne.fr (A.M.); Rafik.benchaabane@fsm.rnu.tn (R.B.C.)

[2] Laboratoire Interdisciplinaire Carnot de Bourgogne (ICB), UMR 6303 CNRS, Université Bourgogne Franche-Comté, 9 av. A. Savary, BP 47870, 21078 Dijon, France; Julien.Boudon@u-bourgogne.fr (J.B.); Lucien.Saviot@u-bourgogne.fr (L.S.)

[3] NANOMISENE Lab, LR16CRMN01, Centre for Research on Microelectronics and Nanotechnology CRMN of Technopark of Sousse, B.P. 334, Sahloul, 4034 Sousse, Tunisia; mosaab.echabaane@gmail.com

[4] Laboratory of Physics of Materials and Nanomaterials Applied at Environment (LaPhyMNE), Faculty of Sciences of Gabes, University of Gabes, 6029 Gabes, Tunisia; omrikarim16@gmail.com

* Correspondence: nmillot@u-bourgogne.fr; Tel.: +33-380-395-937

S1. X Ray Diffraction analysis of $Cu_{0.05}Zn_{0.95}O$ NPs

The X-ray diffraction patterns of $Cu_{0.05}Zn_{0.95}O$ NPs is shown in Figure S1. In addition to peaks relating to ZnO, very low intensity peaks are observed in the XRD patterns with increasing Cu doping up to x=0.05. Specifically, the ($\bar{2}02$) peak that seems to be the most intense. The appearance of these peaks clearly indicates that unreacted Cu^{2+} ions present in the solution gets segregated and formed a secondary phase.

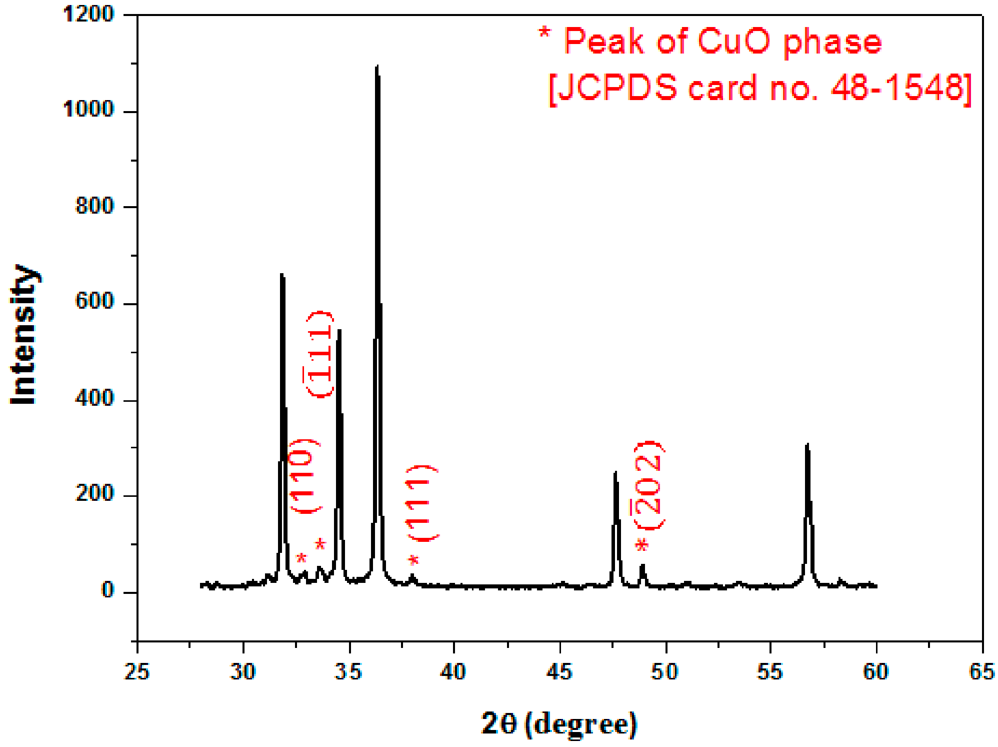

Figure 1. XRD patterns of $Cu_{0.05}Zn_{0.95}O$ NPs.

S2. Example of fit of the Nyquist and Bode diagrams of $Cu_{0.04}Zn_{0.96}O$/ITO

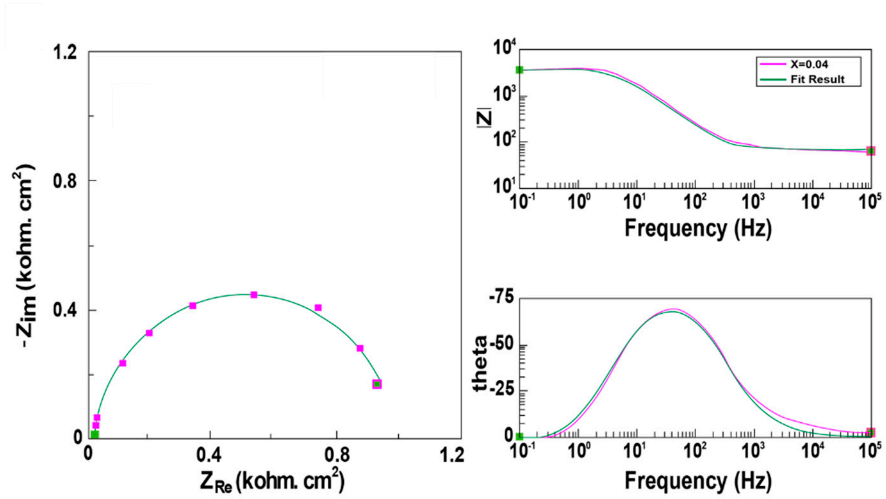

Figure 2. Example of fit of the Nyquist and Bode diagrams of $Cu_{0.04}Zn_{0.96}O$/ITO.

S3. Cyclic voltammograms of $Cu_{0.05}Zn_{0.95}O$ NPs

The cyclic voltammograms of $Cu_{0.05}Zn_{0.95}O$ NPs in the absence and in the presence of glucose are presented in Figure S3. According to the cyclic voltammograms, $Cu_{0.04}Zn_{0.96}O$ NPs were more promising for detecting glucose, due to the presence of sharper redox peaks than $Cu_{0.05}Zn_{0.95}O$ NPs. It seems that the appearance of the secondary CuO phase with increasing Cu doping up to x=0.05, affects the NPs ability to detect glucose.

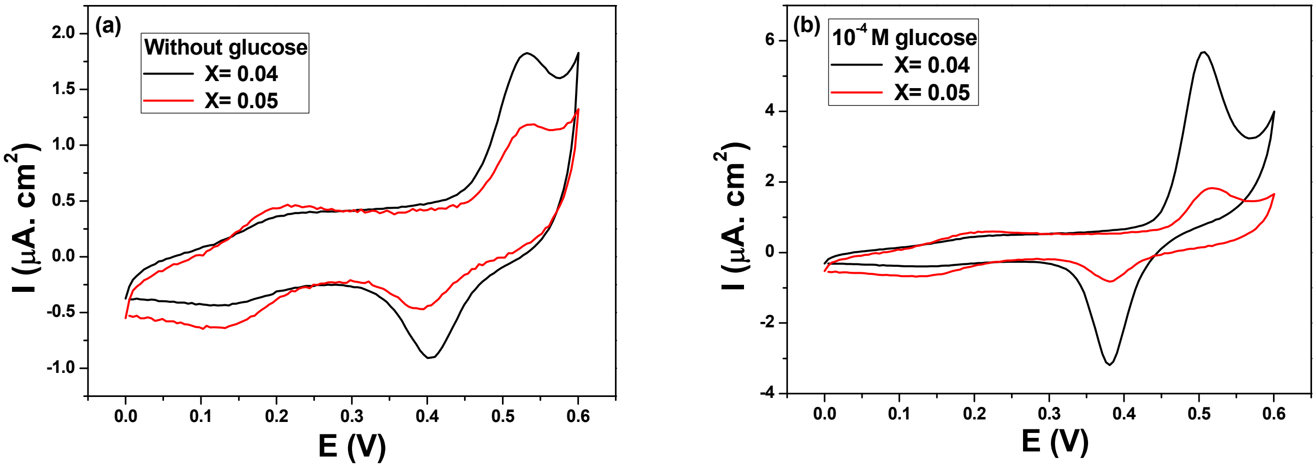

Figure 3. Cyclic voltammograms of $Cu_xZn_{1-x}O$ NPs (x = 0.04 and x = 0.05) in the absence and in the presence of glucose.

S4. Optimization of the polarization potential for EIS measurements:

Usually, the first step for impedance analysis is the optimization of the polarization potential, which depends on the thin film type. Figure S4 shows the variation of Nyquist plots at different potentials ranging from 0 to 0.7 V. The applied AC signal was 10 mV. The frequency range was varied between 100 kHz and 0.1 Hz. As it can be seen, an obvious decrease of the Warburg straight line was observed as the applied potential is increased. In addition, the impedance spectra obtained at 0.7 V presented a semi-circle shape, which facilitates the interpretation of physico-chemical phenomena. For this reason, following impedance studies was performed at a polarization potential of 0.7 V.

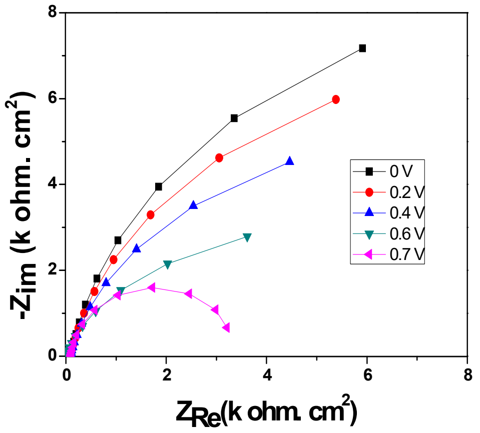

Figure 4. Nyquist plots for ZnO/ITO electrode in 0.1 M NaOH obtained at various applied potential from 0 to 0.7 V, recorded with modulation potential of 10 mV and frequency range from 100 kHz to 0.1 Hz.
